# Effort-Related Upper Extremity Deep Vein Thrombosis: A Case Series of Three Patients With Paget-Schröetter Syndrome Managed in Acute Care Settings

**DOI:** 10.7759/cureus.115886

**Published:** 2026-09-07

**Authors:** Madhura VadlamudiChandrashekar, Jainy Parikh, Hira Sheheryar, Mohiuddin Sharief

**Affiliations:** 1 Medicine, Lancashire Teaching Hospitals NHS Foundation Trust, Preston, GBR; 2 Acute Medicine, Lancashire Teaching Hospital - Royal Preston Hospital, Preston, GBR

**Keywords:** deep vein thrombosis (dvt), effort thrombosis, paget-schröetter syndrome, thoracic outlet, upper limb dvt

## Abstract

Paget-Schröetter syndrome (PSS), also known as effort thrombosis, is a rare form of primary upper extremity deep vein thrombosis (UEDVT) caused by repetitive upper limb activity and/or venous thoracic outlet compression. Delayed recognition may result in pulmonary embolism, post-thrombotic syndrome, and chronic venous obstruction. We present three cases of effort-related UEDVT diagnosed in acute care settings with varying clinical presentations and management strategies.

The first case involved a 78-year-old man who developed acute left upper limb swelling following prolonged gardening activity. The second case concerned a 45-year-old woman who presented with progressive left arm swelling after repetitive shoulder movements during Pilates. The third case involved a 27-year-old male athlete whose initial presentation mimicked a musculoskeletal injury before extensive subclavian and axillary vein thrombosis was identified.

All patients underwent duplex ultrasonography confirming upper extremity venous thrombosis and received therapeutic anticoagulation. One patient was offered catheter-directed thrombectomy but elected conservative treatment. None demonstrated definitive thoracic outlet syndrome on subsequent investigations. Follow-up imaging demonstrated either re-canalization or complete resolution of thrombosis.

These cases highlight the diverse presentations of effort-related UEDVT and emphasize the importance of maintaining a high index of suspicion, even in the absence of obvious swelling or traditional thrombotic risk factors. Early imaging and vascular specialist involvement are essential for optimal outcomes.

## Introduction

Upper extremity deep vein thrombosis (UEDVT) accounts for approximately 5%-10% of all deep vein thromboses and is classified as either primary or secondary [1,2].

Primary UEDVT includes Paget-Schröetter syndrome (PSS), also known as effort thrombosis, which refers to thrombosis of the axillary-subclavian venous system associated with strenuous or repetitive upper limb activity, often in the absence of conventional thrombotic risk factors [3,4]. The condition is thought to result from dynamic compression and repetitive microtrauma of the subclavian vein within the costoclavicular space, leading to endothelial injury, venous stasis, and subsequent thrombosis [3,5]. Although PSS is classically described in young, physically active individuals, it can occur across a broader age range and following a variety of upper limb activities [6,7].

Secondary UEDVT is commonly associated with central venous catheters, malignancy, thrombophilia, or trauma [1,2].

UEDVT may be associated with complications including pulmonary embolism, chronic venous obstruction, and post-thrombotic symptoms [8,9]. We describe three patients presenting to acute medical services with effort-related UEDVT, demonstrating the spectrum of clinical presentation, investigation findings, and management approaches.

## Case presentation

Case 1

A 78-year-old previously independent man presented to the Same Day Emergency Care unit with a one-week history of left arm swelling, heaviness, pain, and visibly prominent superficial veins. Symptoms developed after spending an entire day performing strenuous gardening and digging activities in warm weather, with no other identifiable provoking factor.

He had no history of recent trauma, central venous catheterization, malignancy, previous venous thromboembolism, known thrombophilia, or other recognized provoking factors for UEDVT.

Examination demonstrated edema of the left upper limb with prominent superficial collateral veins extending across the shoulder and upper arm. Peripheral pulses were preserved, and neurological examination was normal. Laboratory investigations were unremarkable apart from an elevated D-dimer of 2.9 mg/L FEU. Duplex ultrasonography demonstrated acute occlusive thrombosis involving the left subclavian and axillary veins with additional non-occlusive thrombus within the brachial veins.

The patient was treated with therapeutic anticoagulation for six months. No invasive intervention was pursued. Symptoms gradually improved during follow-up.

Case 2

A 45-year-old previously fit and well woman presented with progressive pain and swelling of the left upper limb over a two-week period. Symptoms began shortly after participating in a Pilates session involving repetitive rotational shoulder movements. She denied recent trauma, surgery, intravenous cannulation, immobilization, malignancy, or previous thrombotic events.

Clinical examination revealed visible swelling of the left upper limb and prominent superficial collateral veins (Figure 1). There was no erythema or evidence of infection. Distal neurovascular examination was normal.

**Figure 1:**
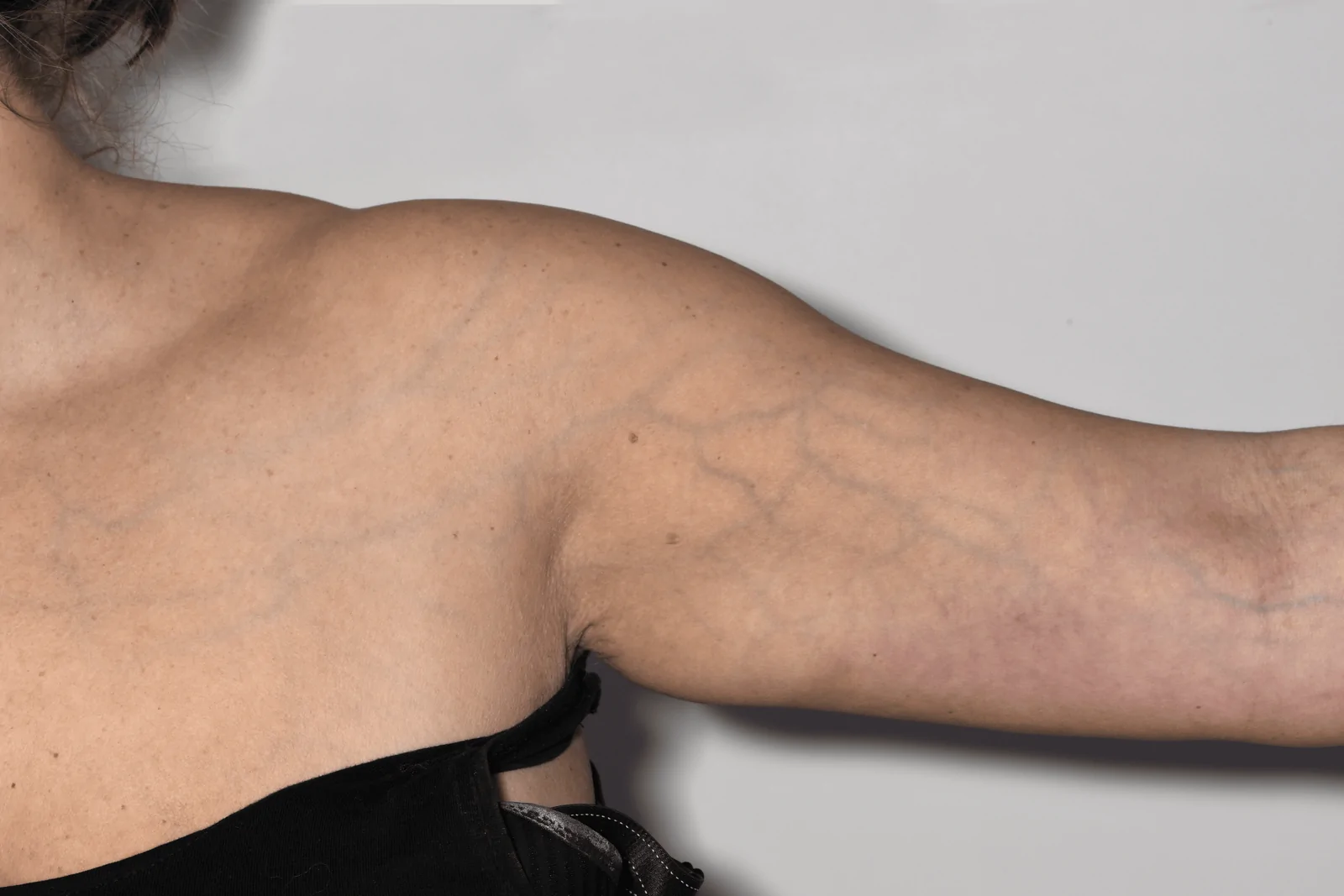
Upper limb swelling and dilated veins

Blood tests demonstrated thrombocytosis and an elevated D-dimer. Duplex ultrasonography confirmed occlusive thrombosis of the left subclavian and basilic veins with patent axillary and brachial veins.

The laboratory findings for all three patients are summarized in Table 1.

**Table 1:** Laboratory findings of the three patients with Paget-Schröetter syndrome CRP: C-reactive protein

Laboratory parameter	Case 1	Case 2	Case 3	Reference range
Hemoglobin (g/L)	147	129	155	115-165
White blood cell count (×10⁹/L)	5.98	8.53	10.01	4.00-11.00
Platelet count (×10⁹/L)	309	489	237	140-440
CRP (mg/L)	1.5	1.1	21.8	0.0-5.0
D-dimer (mg/L FEU)	2.90	1.19	1.82	0.0-0.5

The patient had already commenced apixaban following initial primary care assessment. She was commenced on apixaban 10 mg twice daily for seven days, followed by 5 mg twice daily, in accordance with the recommended treatment regimen for venous thromboembolism.

Vascular surgical review recommended conservative management with anticoagulation, limb elevation, and compression therapy. Further specialist assessment did not identify indications for thrombolysis or thoracic outlet decompression.

At follow-up, she remained clinically stable without evidence of pulmonary embolism or recurrent thrombosis.

Case 3

A 27-year-old male athlete presented to the emergency department with a three-day history of right antecubital fossa pain, arm heaviness, and subjective swelling following strenuous exercise, including hill running and endurance training. His medical history included anxiety and well-controlled asthma.

Initial examination demonstrated no objective swelling, vascular compromise, or neurological deficit. A musculoskeletal injury was suspected, and he was discharged.

Two days later, he re-presented via Same Day Emergency Care because of persistent symptoms and increasingly prominent superficial veins. He reported participation in marathon running and Thai boxing.

Examination again demonstrated symmetrical upper limbs without measurable swelling, erythema, or tenderness (Figure 2). However, D-dimer was elevated at 1.82 mg/L FEU, prompting urgent duplex ultrasonography.

**Figure 2:**
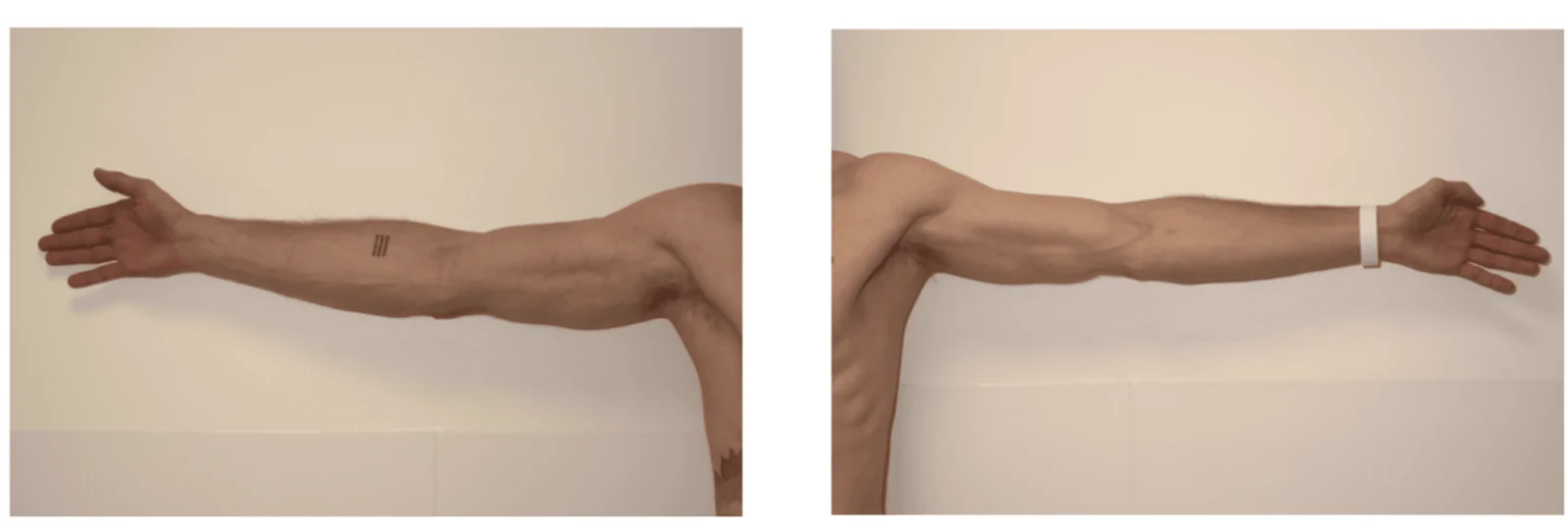
Prominent superficial veins of the right upper limb following strenuous exercise, without significant objective swelling

Imaging revealed extensive acute thrombosis involving the right subclavian and axillary veins with partially occlusive thrombus within the proximal brachial veins (Figure 3 and Figure 4). 

**Figure 3:**
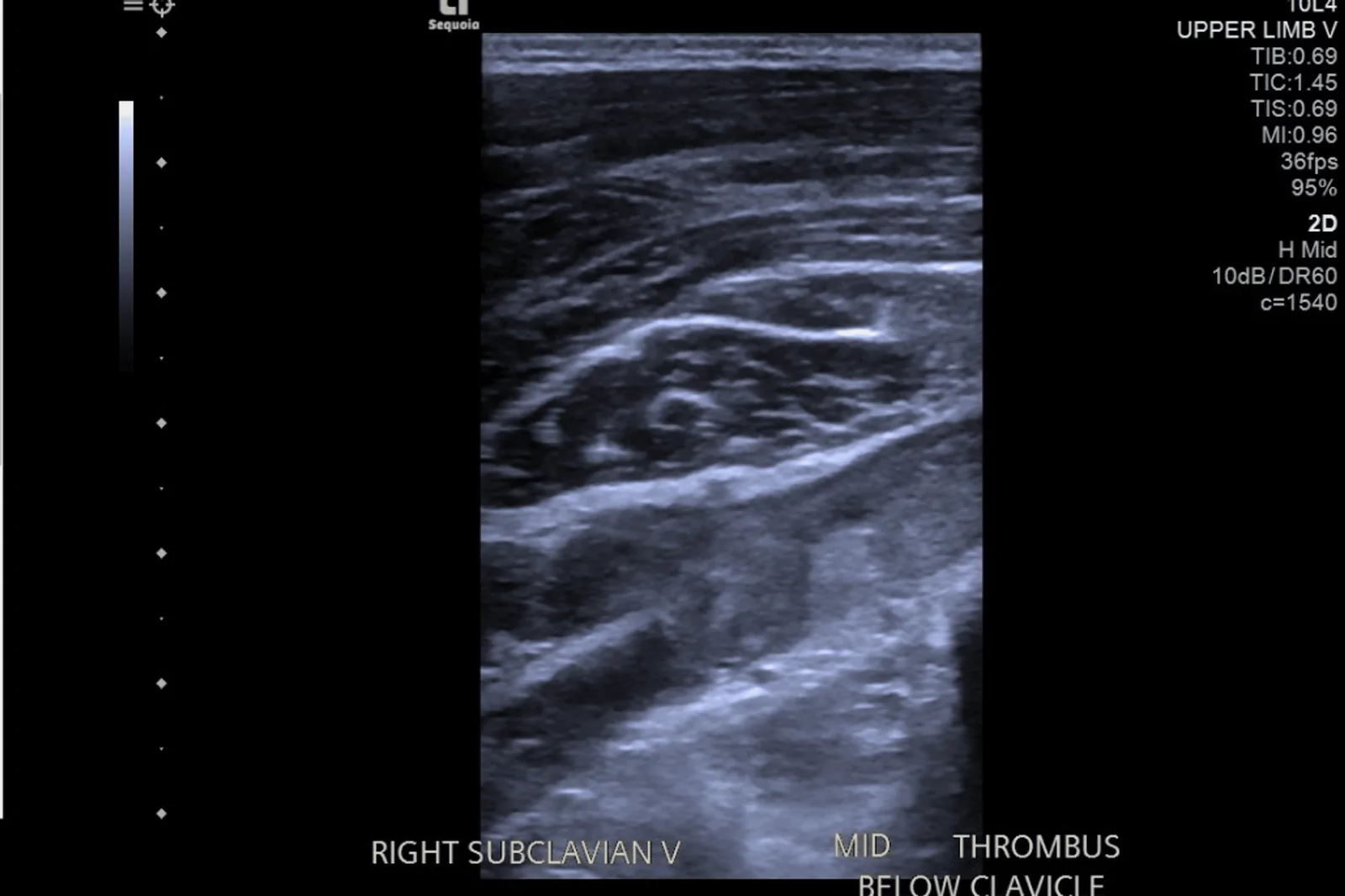
Ultrasound image demonstrating thrombus within the right subclavian vein below the clavicle

**Figure 4:**
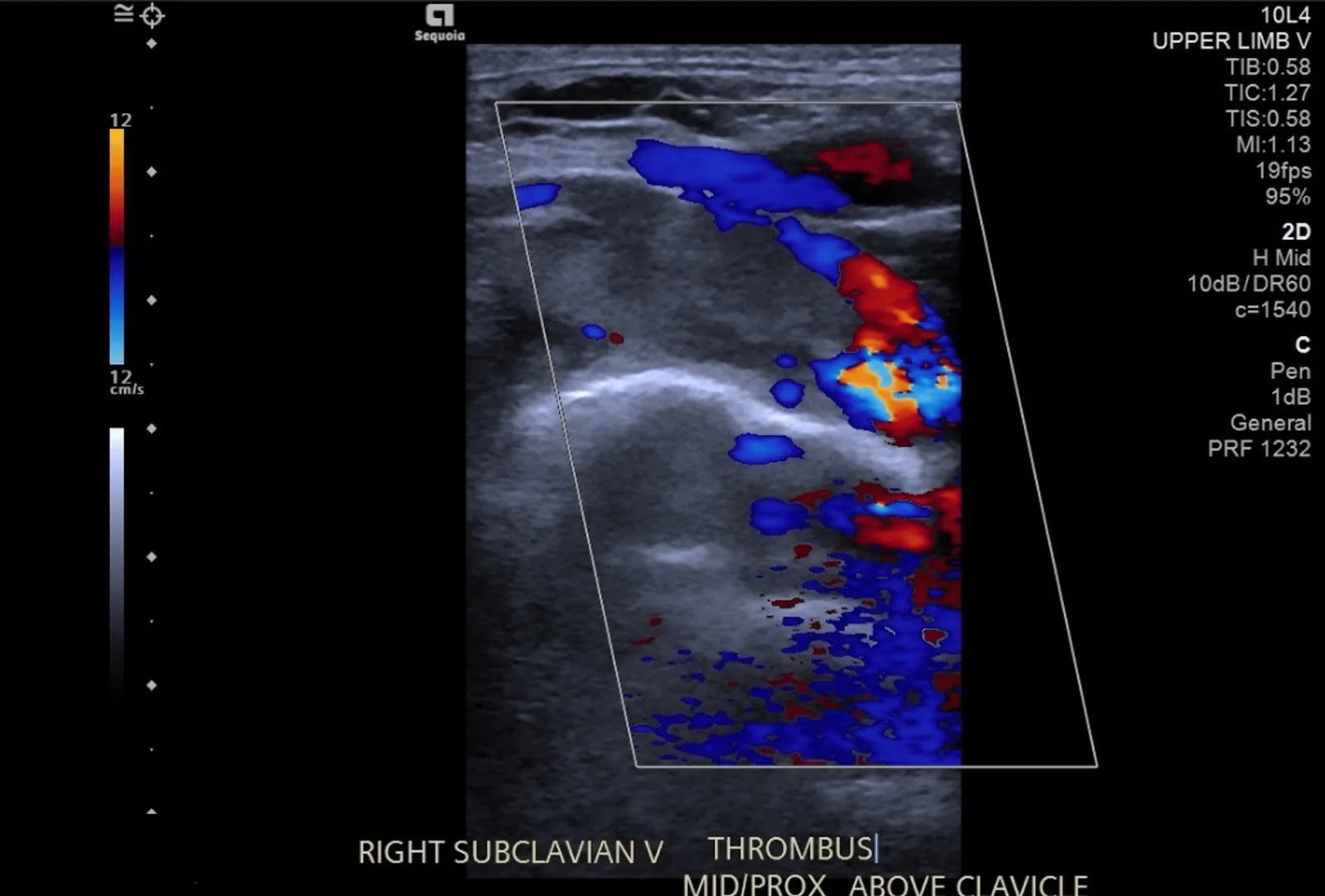
Ultrasound image demonstrating thrombus within the proximal right subclavian vein above the clavicle

Following vascular review, the patient was offered catheter-directed thrombectomy but elected for anticoagulation alone. He was commenced on apixaban 10 mg twice daily for seven days, followed by 5 mg twice daily, in accordance with the recommended treatment regimen for venous thromboembolism, and strenuous upper limb exercise was discontinued.

Subsequent MRI showed no evidence of thoracic outlet syndrome (Figure 5). Repeat ultrasound demonstrated partial re-canalization of the subclavian vein. Thrombophilia screening was negative. Follow-up imaging approximately 18 months later confirmed complete resolution of thrombosis.

**Figure 5:**
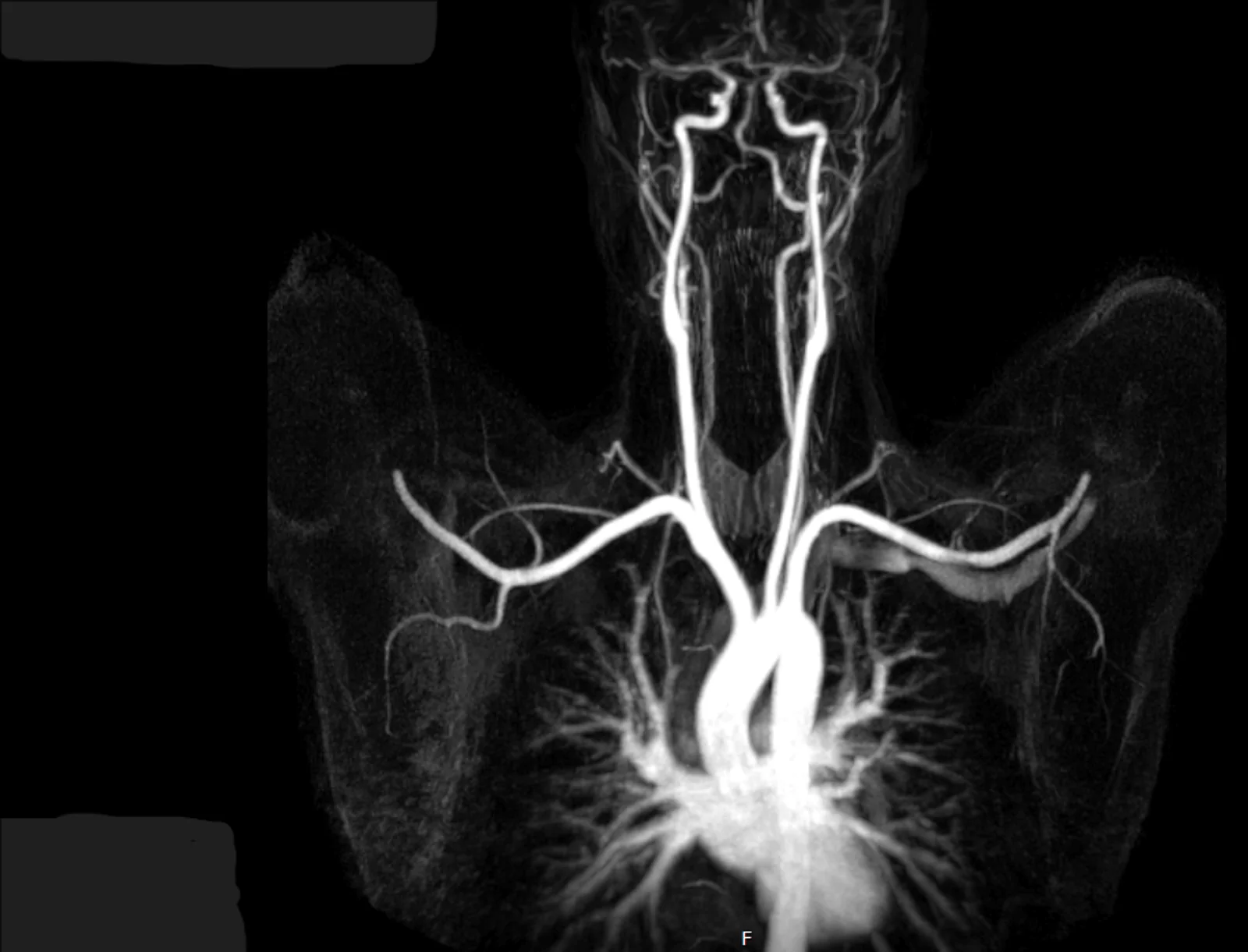
MRI demonstrating no evidence of thoracic outlet syndrome

## Discussion

The three cases presented here demonstrate the varied clinical spectrum of Paget-Schröetter syndrome (PSS) (Table 2). Although PSS is classically described in young male athletes, our patients ranged in age from 27 to 78 years, highlighting that effort-related upper extremity deep vein thrombosis (UEDVT) can occur across a much wider age range. In all three cases, symptoms developed after strenuous or repetitive upper limb activity, consistent with the proposed mechanism of repetitive venous microtrauma leading to thrombosis [3,6,7].

**Table 2:** Clinical presentation, investigations, management, and outcomes of the three patients with Paget-Schröetter syndrome

	Case 1	Case 2	Case 3
Age/sex	78/male	45/female	27/male
Triggers	Gardening/digging	Pilates	Hill running and Thai boxing
Presentation	Left arm pain, swelling, heaviness, prominent veins	Left arm pain, swelling, prominent veins	Arm pain, heaviness, prominent veins, no objective swelling
Ultrasound findings	Left subclavian, axillary, and brachial vein thrombosis	Left subclavian and basilic vein thrombosis	Right subclavian, axillary, and brachial vein thrombosis
Thoracic outlet syndrome	Not demonstrated	Not demonstrated	Negative MRI
Treatment	Anticoagulated (6 months)	Apixaban, compression, conservative management	Offered thrombectomy (declined), apixaban for 6 months
Outcome	Symptoms improved	Stable, no recurrence	Complete thrombus resolution

PSS has traditionally been associated with young male athletes participating in repetitive overhead or strenuous upper limb activities. However, the literature demonstrates a broader range of clinical settings and precipitating activities. Cases have been reported following occupational manual handling and various sporting activities, including throwing sports, as well as in individuals outside the traditionally recognized young athletic population [6,7]. Clinical manifestations may similarly vary from marked limb swelling and prominent collateral veins to isolated pain, heaviness, or subtle venous changes [3,10,11]. This variability is clinically important because the absence of classical swelling does not necessarily exclude extensive axillary-subclavian thrombosis.

Another important finding was the variability in clinical presentation. The first two patients presented with the typical features of upper limb swelling and prominent superficial veins, making the diagnosis relatively straightforward. In contrast, the third patient had only arm pain and heaviness with no objective swelling on initial examination despite extensive thrombosis. This resulted in an initial diagnosis of musculoskeletal injury and illustrates how easily PSS can be overlooked when classical signs are absent [3,10,11]. A raised D-dimer and a low threshold for performing duplex ultrasonography were key to establishing the diagnosis.

Interestingly, none of the patients had evidence of fixed thoracic outlet syndrome on subsequent investigations. The absence of demonstrable anatomical compression does not necessarily exclude an effort-related mechanism, as venous compression may be dynamic or exercise-dependent and may not be evident on investigations performed after the acute thrombotic event [3,5]. This finding has been reported previously and suggests that venous compression may be intermittent, exercise-induced, or no longer demonstrable once the acute thrombotic event has occurred [3,5]. Therefore, the absence of anatomical abnormalities should not exclude the diagnosis when the clinical history is suggestive.

Management of PSS remains an area of ongoing debate. While early catheter-directed thrombolysis followed by thoracic outlet decompression may improve long-term venous patency in carefully selected patients, anticoagulation alone remains an appropriate option for many patients, particularly when presentation is delayed, symptoms are improving, or invasive treatment is declined [4,5,12-14]. In our series, all three patients were managed primarily with anticoagulation, and one patient who was offered catheter-directed thrombectomy chose conservative treatment. All patients had favorable clinical outcomes, with follow-up imaging demonstrating recanalization or complete thrombus resolution.

These cases reinforce the importance of maintaining a high index of suspicion for PSS in any patient presenting with upper limb pain, swelling, heaviness, or prominent superficial veins after strenuous or repetitive arm activity, regardless of age. Prompt duplex ultrasonography and early involvement of vascular specialists are essential to establish the diagnosis, guide management, and minimize the risk of complications such as pulmonary embolism, chronic venous obstruction, and post-thrombotic syndrome [8-11].

## Conclusions

Paget-Schröetter syndrome should be considered in any patient presenting with upper limb pain, swelling, heaviness, or prominent superficial veins following strenuous or repetitive arm activity. Diagnosis may be challenging because examination findings can be subtle or absent despite extensive thrombosis. Duplex ultrasonography remains the first-line diagnostic investigation and should be performed promptly when clinical suspicion exists.

Our case series illustrates that effort-related UEDVT can occur across a wide age range and that anticoagulation alone may result in favorable outcomes in selected patients. Early recognition and specialist vascular assessment remain essential to optimize outcomes and prevent long-term complications.
